# Development and validation for multifactor prediction model of sudden sensorineural hearing loss

**DOI:** 10.3389/fneur.2023.1134564

**Published:** 2023-05-18

**Authors:** Chaojun Zeng, Yunhua Yang, Shuna Huang, Wenjuan He, Zhang Cai, Dongdong Huang, Chang Lin, Junying Chen

**Affiliations:** ^1^Department of Otorhinolaryngology Head and Neck Surgery, Fujian Institute of Otorhinolaryngology, The First Affiliated Hospital, Fujian Medical University, Fuzhou, China; ^2^National Regional Medical Center, Binhai Campus of the First Affiliated Hospital, Fujian Medical University, Fuzhou, China; ^3^Department of Otorhinolaryngology Head and Neck Surgery, First Hospital of Putian City, Putian, Fujian, China; ^4^Department of Otolaryngology, Fujian Provincial Geriatric Hospital, Fuzhou, China; ^5^Department of Clinical Research and Translation Center, The First Affiliated Hospital, Fujian Medical University, Fuzhou, China; ^6^Clinical Laboratory, Fujian Provincial Hospital South Branch, Fuzhou, China; ^7^Central Laboratory, Key Laboratory of Radiation Biology of Fujian Higher Education Institutions, The First Affiliated Hospital, Fujian Medical University, Fuzhou, China

**Keywords:** sudden sensorineural hearing loss, prediction, nomogram, thrombin time, red blood cell, granulocyte lymphocyte ratio

## Abstract

**Background:**

Sudden sensorineural hearing loss (SSNHL) is a global problem threatening human health. Early and rapid diagnosis contributes to effective treatment. However, there is a lack of effective SSNHL prediction models.

**Methods:**

A retrospective study of SSNHL patients from Fujian Geriatric Hospital (the development cohort with 77 participants) was conducted and data from First Hospital of Putian City (the validation cohort with 57 participants) from January 2018 to December 2021 were validated. Basic characteristics and the results of the conventional coagulation test (CCT) and the blood routine test (BRT) were then evaluated. Binary logistic regression was used to develop a prediction model to identify variables significantly associated with SSNHL, which were then included in the nomogram. The discrimination and calibration ability of the nomogram was evaluated by receiver operating characteristic (ROC), calibration plot, and decision curve analysis both in the development and validation cohorts. Delong’s test was used to calculate the difference in ROC curves between the two cohorts.

**Results:**

Thrombin time (TT), red blood cell (RBC), and granulocyte–lymphocyte ratio (GLR) were found to be associated with the diagnosis of SSNHL. A prediction nomogram was constructed using these three predictors. The AUC in the development and validation cohorts was 0.871 (95% CI: 0.789–0.953) and 0.759 (95% CI: 0.635–0.883), respectively. Delong’s test showed no significant difference in the ROC curves between the two groups (*D* = 1.482, *p* = 0.141).

**Conclusion:**

In this study, a multifactor prediction model for SSNHL was established and validated. The factors included in the model could be easily and quickly accessed, which could help physicians make early diagnosis and clinical treatment decisions.

## Introduction

Sudden sensorineural hearing loss (SSNHL) is defined as a rapid hearing loss of at least 30 dB at three consecutive frequencies within 72 h (1). As an otological emergency, SSNHL has an annual incidence of 5–30/100,000 cases, with an increasing trend worldwide (1). Only 4.47–15% of cases of SSNHL can be identified with a final diagnosis in the acute stage (2), such as labyrinthine hemorrhage (3) or vascular events (4). The prognosis of SSNHL is poor due to unclear pathophysiology, delayed diagnosis, and treatment. If the disease can be predicted in advance, it will help to give timely treatment and improve the therapeutic effect of SSNHL. Therefore, such prediction models are very important to SSNHL.

Ischemia and hypoxia caused by the alteration of blood flow are important causes of SSNHL (5). Several routine hematological parameters have been identified as prognostic factors, including platelet-to-lymphocyte ratio (PLR), neutrophil–lymphocyte ratio (NLR), and fibrinogen–albumin ratio (FAR) (6–9). However, the relationship between thrombin time (TT), red blood cell (RBC), granulocyte lymphocyte ratio (GLR), and SSNHL is not fully understood. It is worth noting that most of these studies have only predicted the prognosis of SSNHL, and these prognosis models were developed using data from single-center data with different clinical outcome settings leading to different conclusions. Even if several studies previously investigated diagnostic markers (10–12), few studies focused on prediction models for SSNHL.

Therefore, the purpose of this study was to develop a prediction model in the development cohort and verify its reliability in the validation cohort, thus establishing a promising prediction model for SSNHL.

## Methods

### Study participants

**Figure 1:**
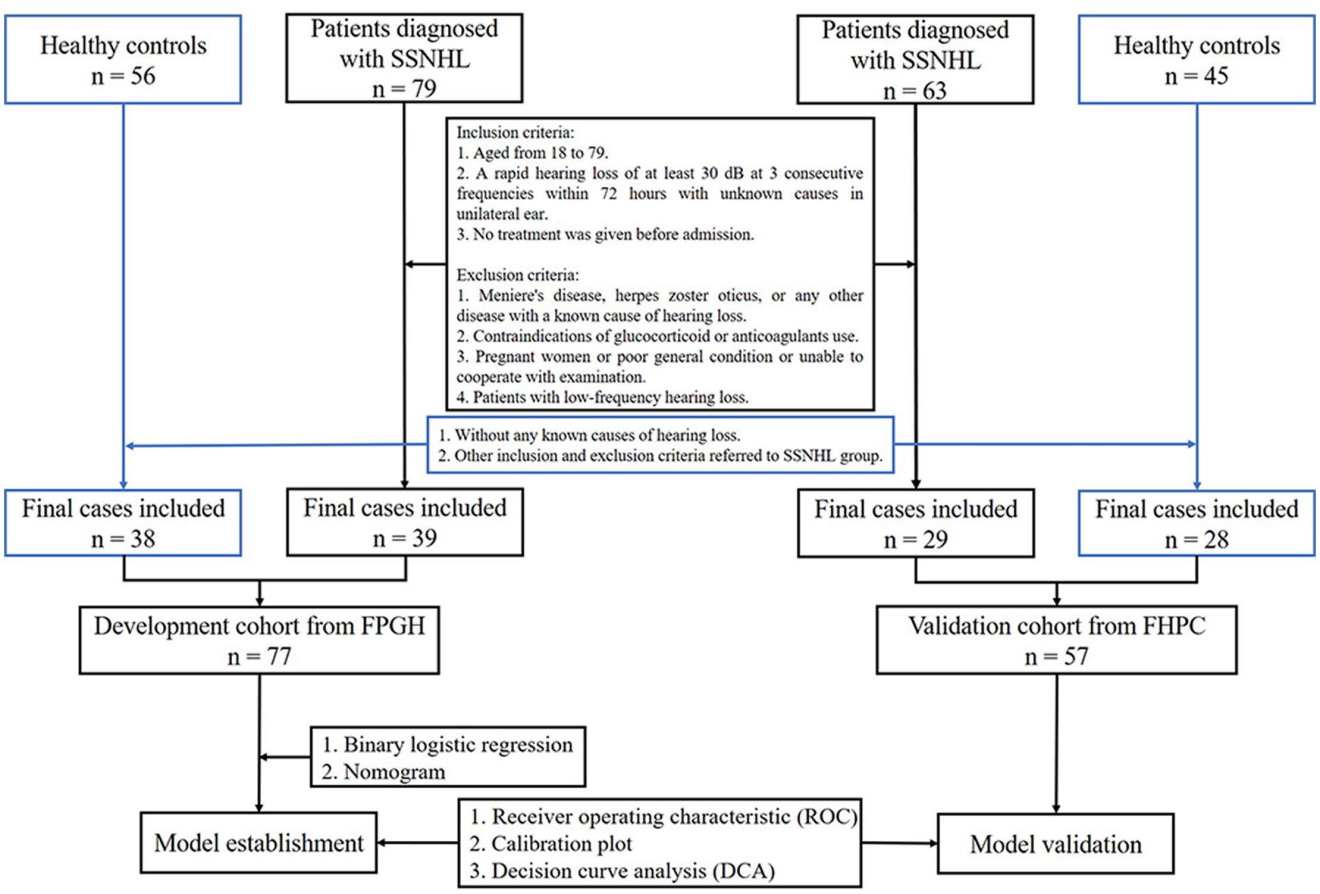
Flow chart (FPGH, Fujian Provincial Geriatric Hospital; FHPC, First Hospital of Putian City).

SSNHL refers to a sudden occurrence of sensorineural hearing loss of unknown etiology within 72 h with at least three consecutive frequency losses of 30 dB (1). In the development cohort, Fujian Provincial Geriatric Hospital recruited 39 patients diagnosed with SSNHL and 38 adults who were free of any ear disease, cancer, or any other blood disease in the physical examination as healthy controls from January 2018 to December 2021. All participants ranged in age from 18 to 79 years and underwent conventional coagulation test (CCT) and blood routine test (BRT). We excluded pregnant women and patients who are taking anticoagulant drugs, as well as patients with blood diseases, Meniere’s disease, herpes zoster oticus, or any other disease with a known cause of hearing loss. Because low-frequency hearing loss is one of the symptoms of Meniere’s disease, patients with low-frequency hearing loss were also excluded. At the same time, 57 adults (29 SSNHL patients and 28 healthy controls) from First Hospital of Putian City were incorporated into the validation cohort. The exclusion criteria were the same as the development cohort as depicted in Figure 1.

This study was reviewed and approved by the institutional review board and ethics committee of Fujian Geriatric Hospital (Ethics Committee No.2020-03-01). All the methods were carried out in accordance with the relevant guidelines and regulations. Informed consent was obtained from all participants.

### Data collection

Detailed medical history, which includes details of baseline characteristics (including age, gender, hypertension, diabetes, hypertriglyceridemia, hypercholesterolemia, and autoimmune disease), clinical characteristics (including affected side), accompanying symptoms (such as tinnitus, vertigo, headache, and dizziness), was obtained from all participants.

### Clinical and other measurements

Hearing loss was determined by pure-tone audiometry (Conera, Denmark) before and after treatment. Pure-tone average (PTA) was calculated as the average of thresholds (dB HL) at seven frequencies of 0.125, 0.25, 0.5, 1, 2, 4, and 8 kHz. The coagulation function parameters were measured by an automatic coagulation analyzer (Sysmex CS5100 or CS2000i, Japan), and the blood routine parameters were measured by a fully automatic hematology analyzer (Sysmex XN 1000 or CN3000, Japan).

### Statistical analysis

*Post hoc* assessments of sample size were performed. In the development cohort, 39 samples in the positive group and 38 samples in the negative group were tested using a two-sided z-test between the area under the receiver operating characteristic (ROC) curve (AUC) under the null hypothesis of 0.500 and an AUC at the alternative hypothesis of 0.871, achieving>99% power at a significance level of 0.050 and a difference of 0.371 was detected. In the validation cohort, 29 samples in the positive group and 28 samples in the negative group achieved 95% power and the detected difference was 0.259.

Continuous variables were expressed as mean ± standard deviation (SD), while categorical variables were presented as frequencies and percentages (%). To compare the difference between groups, the Chi-square test or the Fisher exact test was used for categorical variables and the *t*-test was used for continuous variables.

The prediction model for the risk of SSNHL was established based on the existing information. The steps are as follows:

First, the baseline characteristics of the control group and SSNHL group in the development cohort were balanced, and the 12 items of CCT and BRT parameters were initially selected for candidate predictors. Second, the potential predictors with *p* < 0.05 in the univariate analyses were selected to be included in a multivariate logistic model. Third, a backward step-down selection process was performed by a threshold of *p* < 0.05 to establish a parsimonious model, and a nomogram was formulated in the training cohort. Fourth, the discriminative ability, predictive accuracy, and clinical application value of the model in the training cohort were assessed using a ROC curve, calibration plot, and decision curve analysis (DCA). Finally, the external validity of model performance was assessed in the validation cohort, and Delong’s test was conducted to compare the ROC curves of the development cohort and the validation cohort.

The statistical software used in this study includes SPSS software version 17.0 (IBM) and R software (version 4.2.1). The *p-*values less than 0.05 were considered statistically significant in each statistical analysis.

## Results

### Baseline characteristics, blood CCT, and BRT parameters in the development and validation cohort

In total, 134 participants were enrolled, 77 in the development cohort and 57 in the validation cohort. The baseline characteristics, CCT, and BRT parameters are detailed in Table 1. Simply, the participants comprised the development cohort (28 women and 49 men) with a median age of 49.57 years and the validation cohort (27 women and 28 men) with a median age of 47.75 years. Both cohorts were comparable not only in terms of age, gender, hypertension, diabetes, hypertriglyceridemia, hypercholesterolemia, and autoimmune disease but also hearing loss, tinnitus, vertigo, dizziness, and headache. The probability of all the basic characteristics in the development cohort was similar to the validation cohort except for hypertension (*p* = 0.027).

**Table 1:** Baseline characteristics, CCT, and BRT parameters in development and validation cohorts.

Variables	Development cohort (*n* = 77)	Validation cohort (*n* = 57)	*χ*^2^ or *t* value	*p*
Age	49.57 ± 12.105	47.75 ± 11.350	0.882	0.379
Male	49(63.6%)	28(49.1%)	2.823	0.093
Hypertension	18(23.4%)	5(8.8%)	4.914	**0.027**
Diabetes	4(5.2%)	3(5.3%)	–	1.000^#^
Hypertriglyceridemia	18(23.4%)	20(35.1%)	2.211	0.137
Hypercholesterolemia	39(50.6%)	28(49.1%)	0.031	0.861
Autoimmune disease	2(2.6%)	0(0%)	–	0.507^#^
Headache	1(1.3%)	0(0%)	–	1.000^#^
Dizziness	4(5.2%)	0(0%)	–	0.136^#^
Vertigo	9(11.7%)	10(17.5%)	0.923	0.337
Hearing loss	39(50.6%)	29(50.9%)	0.001	0.979
Tinnitus	37(48.1%)	27(47.4%)	0.006	0.938
PT	10.90 ± 0.697	10.42 ± 0.555	4.302	**<0.001**
INR	0.95 ± 0.064	0.91 ± 0.049	4.026	**<0.001**
APTT	27.72 ± 3.216	25.03 ± 2.601	5.178	**<0.001**
TT	18.39 ± 1.873	17.39 ± 0.787	4.212	**<0.001**
FIB	2.68 ± 0.526	2.83 ± 0.574	−1.522	0.130
WBC	6.69 ± 2.279	6.77 ± 2.111	−0.205	0.838
RBC	4.82 ± 0.549	4.72 ± 0.517	1.038	0.301
Platelet	241.32 ± 58.294	246.18 ± 59.918	−0.471	0.639
Granulocyte	4.20 ± 2.013	4.26 ± 1.969	−0.153	0.878
Lymphocyte	1.94 ± 0.661	2.00 ± 0.700	−0.557	0.578
Monocyte	0.41 ± 0.160	0.53 ± 1.178	−0.850	0.397
GLR	2.40 ± 1.516	2.59 ± 2.173	−0.603	0.548

CCT, Conventional Coagulation Test; BRT, Blood Routine Test; PT, Prothrombin Time; INR, International Normalized Ratio; APTT, Activated Partial Thromboplastin Time; TT, Thrombin Time; FIB, Fibrinogen; WBC, White Blood Cell; RBC, Red Blood Cell; GLR, Granulocyte Lymphocyte Ratio. Clinical characteristics were analyzed as a classified variable; all CCT, BRT parameters, and age were analyzed as continuous variables. Data were exhibited with Mean ± SD or n (%).

^#^Fisher’s exact test.The bold values means *p* < 0.05.

Five coagulation function parameters and seven blood routine test parameters were within the normal range in both cohorts. The development cohort exhibited a significantly prolonged PT, INR, TT, and APTT compared with the validation cohort (all *p* < 0.001). Fibrinogen (FIB) in the development cohort was lower but had no statistical difference (*p* = 0.130) than in the validation cohort. The seven blood routine test parameters showed no statistical difference in both cohorts (all *p* > 0.05).

### Factors selection for SSNHL prediction model construction in the development cohort

In the development cohort, all baseline characteristics were similar (*p* > 0.05) except for the SSNHL-specific characteristics, including hearing loss (*p* < 0.001), tinnitus (*p* < 0.001), vertigo (*p* = 0.002), in control and SSNHL group (Table 2). The parameters in CCT and BRT, including PT, INR, TT, RBC, granulocyte, lymphocyte, monocyte, and granulocyte to Lymphocyte ratio (GLR), were significantly different (all *p* < 0.05) between the control and the SSNHL group (Table 2).

**Table 2:** Baseline characteristics, CCT and BRT parameters of control and SSNHL group in the development cohort.

Variables	Control (*n* = 38)	SSNHL (*n* = 39)	*χ*^2^ or *t* value	*p*
Age	47.84 ± 11.173	51.26 ± 12.869	−1.242	0.218
Male	26 (68.4)	23 (59.0)	0.742	0.389
Hypertension	9 (23.7)	9 (23.1)	0.004	0.950
Diabetes	2 (5.3)	2 (5.1)	–	1.000
Hypertriglyceridemia	10 (26.3)	8 (20.5)	0.362	0.547
Hypercholesterolemia	18 (47.4)	21(53.8)	0.323	0.570
Autoimmune disease	0 (0.0)	2 (5.1)	–	0.494^#^
Headache	1 (2.6)	0 (0.0)	–	0.494^#^
Dizziness	1 (2.6)	3 (7.7)	–	0.615^#^
Vertigo	0 (0.0)	9 (23.1)	–	**0.002**
Hearing loss	0 (0.0)	39 (100.0)	77.000	**<0.001**
Tinnitus	1 (2.6)	36 (92.3)	62.006	**<0.001**
PT	10.72 ± 0.549	11.08 ± 0.782	−2.356	**0.021**
INR	0.93 ± 0.051	0.96 ± 0.070	−2.581	**0.012**
APTT	27.82 ± 1.934	27.62 ± 4.126	0.274	0.785
TT	17.71 ± 2.248	19.05 ± 1.091	−3.039	**0.002**
FIB	2.69 ± 0.500	2.67 ± 0.557	0.155	0.877
WBC	6.22 ± 1.804	7.16 ± 2.602	−1.851	0.069
RBC	4.99 ± 0.453	4.65 ± 0.588	2.840	**0.006**
Platelet	248.68 ± 56.497	234.15 ± 59.846	1.095	0.277
Granulocyte	3.58 ± 1.582	4.81 ± 2.212	−2.816	**0.006**
Lymphocyte	2.10 ± 0.531	1.78 ± 0.738	2.226	**0.029**
Monocyte	0.37 ± 0.092	0.46 ± 1.199	−2.336	**0.023**
GLR	1.76 ± 0.979	3.02 ± 1.782	−4.020	**<0.001**

CCT, Conventional Coagulation Test; BRT, Blood Routine Test; PT, Prothrombin Time; INR, International Normalized Ratio; APTT, Activated Partial Thromboplastin Time; TT, Thrombin Time; FIB, Fibrinogen; WBC, White Blood Cell; RBC, Red Blood Cell; GLR, Granulocyte Lymphocyte Ratio. Clinical characteristics were analyzed as a classified variable; all CCT, BRT parameters, and age were analyzed as continuous variables. Data were exhibited with Mean ± SD or n (%).

^#^Fisher’s exact test.The bold values means *p* < 0.05.

The factors (*p* < 0.05) were incorporated for the predicted model using logistic regression analysis among the abovementioned CCT and BRT factors. TT (OR = 1.515, 95% CI: 1.031–2.225, *p* = 0.034), RBC (OR = 0.141, 95% CI: 0.039–0.507, *p* = 0.003), and GLR (OR = 3.142, 95% CI: 1.587–6.220, *p* = 0.001) in model 2 can be used for SSNHL prediction model construction (Table 3).

**Table 3:** Different logistic regression models for assessing the diagnostic factors of SSNHL.

Variables	Model 1			Model 2		
	*B*	OR (95% CI)	*p*	*B*	OR (95% CI)	*p*
PT	0.812	2.252(1.103–4.598)	**0.026**	–	–	–
INR^$^	0.975	2.650(1.198–5.863) ^$^	**0.016**	–	–	–
TT	0.484	1.623(1.168–2.256)	**0.004**	0.415	1.515(1.031–2.225)	**0.034**
RBC	−1.244	0.288(0.114–0.730)	**0.009**	−1.958	0.141(0.039–0.507)	**0.003**
Granulocyte	0.373	1.452(1.088–1.938)	**0.011**	–		–
Lymphocyte	−0.856	0.425(0.189–0.953)	**0.038**	–	–	–
Monocyte^$^	0.037	1.458(1.031–2.062) ^$^	**0.033**	–	–	–
GLR	0.979	2.662(1.475–4.807)	**0.001**	1.145	3.142(1.587–6.220)	**0.001**
Constant	–	–	–	−0.731	0.481	–

OR, Odds Ratio; CI, Confidence Interval; PT, Prothrombin Time; INR, International Normalized Ratio; TT, Thrombin Time; RBC, Red Blood Cell; GLR, Granulocyte Lymphocyte Ratio. Model 1, unadjusted; Method = Enter. Model 2, adjusted for all factors that *p* < 0.05 in development cohort at baseline; Method = Backward Stepwise (Wald).

$ Data used for Logistic Regression was 10×.The bold values means *p* < 0.05.

### Construction of the prediction risk model

The three candidate factors (TT, RBC, and GLR) were chosen to construct the prediction risk model. According to the logistic regression derived β coefficients, an individual’s risk of SSNHL might be calculated as follows:


$$\begin{array}{l} P\;\left(\mathrm{SSNHL}\right)=\\ \;\;\;\;\;\;\;\;\;1/\left[1+e^{\left(0.731-\mathrm{TT}\times 0.415+\mathrm{RBC}\times 1.958-\mathrm{Granulocyte}/\mathrm{Lymphocyte}\times 1.145\right)}\right] \end{array}$$

**Figure 2:**
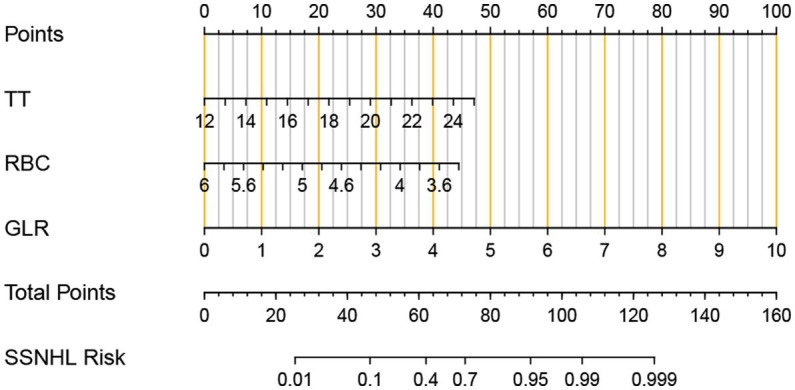
Nomogram for predicting the risk of an individual adult sudden sensorineural hearing loss (SSNHL). The values of thrombin time (TT), red blood cell (RBC), granulocyte lymphocyte Ratio (GLR), and points are acquired from each variable axis. The total points on the axis are the sum values of these three factors, which can predict the SSNHL risk.

To evaluate the prediction model more readable and convenient, a nomogram was constructed, as shown in Figure 2.

### Validation of the risk model

**Figure 3:**
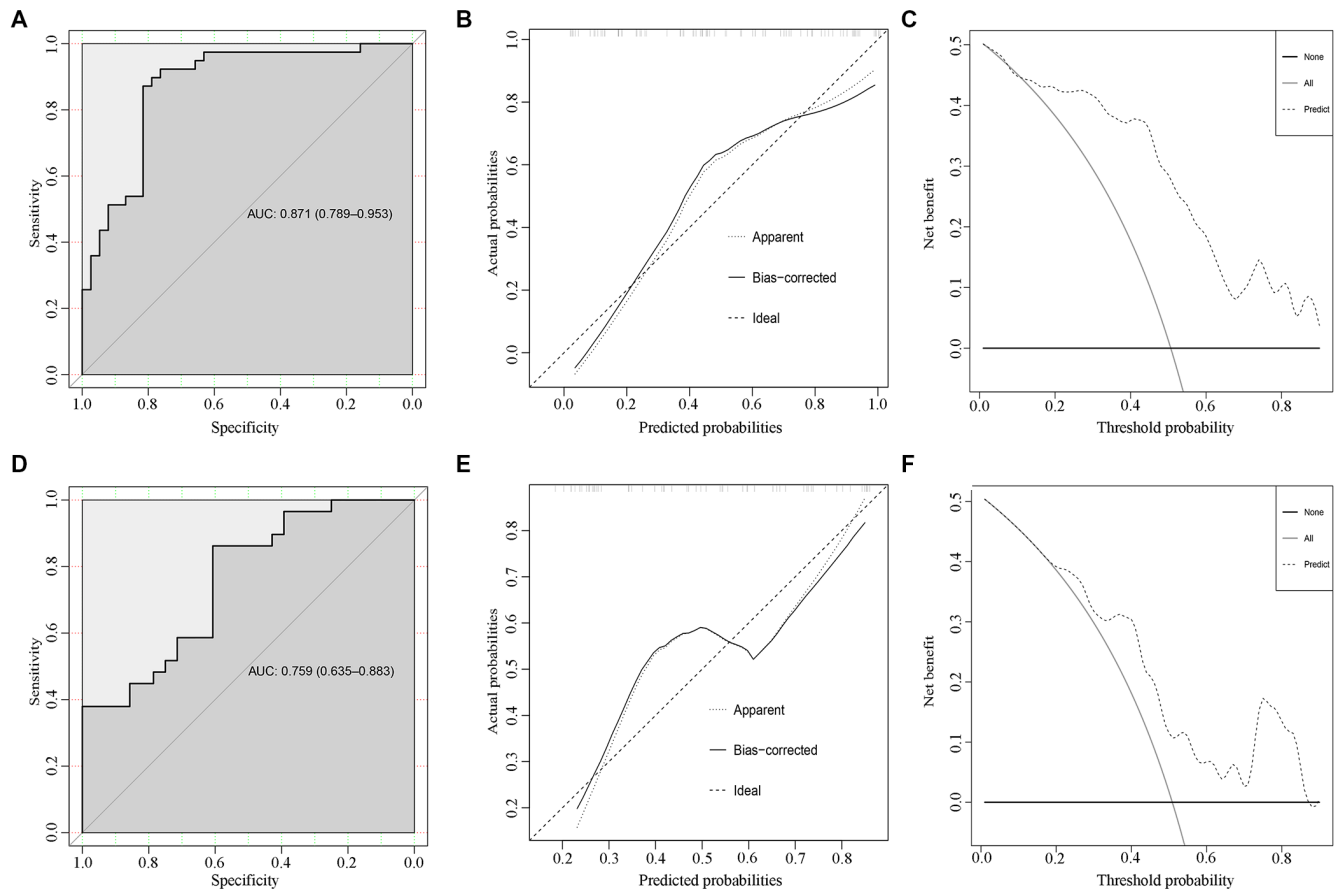
Internal and external validations for the SSNHL prediction model. The ROC, calibration plots, and decision curve analysis for the predicting model in the development cohort **(A–C)** and the validation cohort **(D–F)**.

Based on the development cohort, the ROC analysis was significantly different between the control group and the SSNHL group (*p* < 0.001) (Figure 3A). The AUC value of the prediction model was 0.871 (95% CI: 0.789–0.953). The calibration plot is shown in Figure 3B, and DCA is shown in Figure 3C.

The SSNHL prediction model was evaluated in the validation cohort. The ROC analysis was significantly different between the two groups (*p* < 0.001) (Figure 3D). The AUC value of the prediction model was 0.759 (95% CI: 0.635–0.883).

Delong’s test for two ROC curves did not find a significant difference between the AUC in the development cohort and the validation cohort (*D* = 1.482, *p* = 0.141). The calibration plot is shown in Figure 3E, and DCA is shown in Figure 3F.

## Discussion

The diagnosis of SSNHL is mainly based on the audiogram; however, a subjective audiogram alone cannot accurately diagnose SSNHL, and may also misdiagnose prognosis. In this study, a prediction model of SSNHL was established via binary logistic regression and verified externally. TT, RBC, and GLR were identified as the diagnostic factors for SSNHL and used to build a reliable nomogram. Nomogram has been shown to predict SSNHL in both the development and the validation cohorts and is strongly recommended for clinical use.

In this study, TT was considered to be a risk factor for SSNHL. Prolonged TT was seen in diseases with reduced plasma fibrinogen, such as disseminated intravascular coagulation (12) and dysfibrinogenemia (13). A previous study has shown that dysfunction of inner ear microcirculation plays an important role in SSNHL (14); however, due to the lack of effective evidence-based evidence, the use of anticoagulants has not reached an international consensus (11). Labyrinthine hemorrhage has also been reported as a potential factor for SSNHL (3); meanwhile, oral anticoagulants may interfere with microcirculation in the inner ear by influencing the viscosity of the plasma (15). Our data showed that TT was prolonged before treatment in the SSNHL group. Therefore, TT should be paid more attention to avoid bleeding events or aggravating the condition in older patients with SSNHL or patients who are taking anticoagulants.

RBC has been identified as a risk factor for myometrial invasion in endometrioid endometrial carcinoma patients with metabolic syndrome (16) and as a significant predictor of the risk of adverse cardiovascular events (17). Studies have indicated that the cochlea is more susceptible to hypoxia at high frequencies in rats (18), and hypoxia can impair hearing function in patients with chronic obstructive pulmonary disease (19). In this study, low RBC count is a risk factor for SSNHL and an indicator of blood viscosity. A low RBC count means reduced blood viscosity and the use of anticoagulants should be carefully considered in patients with SSNHL. Furthermore, the labyrinthian artery is the only small-diameter artery in the cochlea, and the decreased RBC count will lead to the reduction of oxygenation (20), which may aggravate cochlear ischemia and hypoxia, then causes SSNHL or exacerbate the condition.

GLR can be regarded as a reliable indicator to predict infectious complications after gastrectomy (21) or systemic spread of streptococcus pyogenes with acute skin infection (22), which is associated with inflammatory reactions. It suggested that increased GLR can be used as a predicted marker of SSNHL. The mechanism of SSNHL is associated with inflammation, which is well demonstrated by steroid therapy (23). Increased granulocyte and/or decreased lymphocyte (especially viral infection) will lead to increased GLR in the acute inflammation phase of patients with SSNHL. Therefore, GLR as a predictor of SSNHL is reliable and necessary.

In this study, after excluding not statistically significant variables, a prediction model of SSNHL was established by combining TT, RBC, and GLR, and visualized by nomogram. In the development and validation cohorts, the AUC values of the prediction model were 0.871 (95% CI: 0.789–0.953) and 0.759 (95% CI: 0.635–0.883), respectively, showing a certain predictive ability. Meanwhile, the calibration plot and DCA show good performance in the development and validation cohorts. However, it is difficult for all instruments in both centers to be the same. It has been found that the performance of the instruments is stable, and the measurement reference values are the same in both centers. In addition, the highly consistent results between the two centers further indicate the stability of our model. It is also limited by the retrospective design and small sample size, and a prospective cohort study with a larger sample size is needed to further refine this study. Nevertheless, we believe that the prediction model and nomogram will play an important role in guiding the clinical diagnosis and management of SSNHL.

## Conclusion

In this study, a prediction model and nomogram of SSNHL were established with good discrimination and calibration and were helpful for clinicians to diagnose SSNHL timely and to take reasonable treatment decisions.

## Data availability statement

The datasets presented in this article are not readily available because of ethical and privacy restrictions. Requests to access the datasets should be directed to the corresponding authors

## Ethics statement

The studies involving human participants were reviewed and approved by the Institutional Ethics Committee of Fujian Geriatric Hospital (Ethics Committee No. 2020-03-01). Written informed consent to participate in this study was provided by the patient/participants or patient/participants legal guardian/next of kin. Written informed consent was obtained from the individual(s) and/or minor(s)' legal guardian/next of kin for the publication of any potentially identifiable images or data included in this article.

## Author contributions

CZ and JC were involved in study conception and design, designed the experiment, analyzed the data, drafted the manuscript, discussed results, and revised and finalized the manuscript. YY performed the SSNHL diagnosis, collected the data, evaluated the treatment outcome, and discussed the results. SH analyzed the data and confirmed the statistical results. WH collected the data from physical examination persons. ZC and DH performed the SSNHL diagnosis, collected the data, and evaluated the treatment outcome. CL was involved in study conception and design, discussed the results, and revised and finalized the manuscript. All authors approved the final copy of this manuscript.

## Funding

This study was supported by the Natural Science Foundation of Fujian Province (grant nos: 2022J01215 and 2020J01097) and the Research Project of the Treatment of Auditory System Dysfunction in Aging Population (grant no: 2020YFC2005204).

## Conflict of interest

The authors declare that the research was conducted in the absence of any commercial or financial relationships that could be construed as a potential conflict of interest.

## Publisher’s note

All claims expressed in this article are solely those of the authors and do not necessarily represent those of their affiliated organizations, or those of the publisher, the editors and the reviewers. Any product that may be evaluated in this article, or claim that may be made by its manufacturer, is not guaranteed or endorsed by the publisher.
